# Machine-Learning-Based Prediction and Risk for Possible Sarcopenia Among Community-Dwelling Older Adults

**DOI:** 10.1093/geroni/igaf122.2374

**Published:** 2025-12-31

**Authors:** Sooyoung Kwon, Gwang Suk Kim, Layoung Kim, Namhee Kim

**Affiliations:** Graduate School of Yonsei University, Seoul, Republic of Korea; College of Nursing, Yonsei University, Seoul, Republic of Korea; Department of Nursing, The University of Suwon, Hwaseong, Republic of Korea; Yonsei University, Wonju, Republic of Korea

## Abstract

To improve the health and quality of life of older adults, it is important to provide simple, accurate healthcare services that prevent sarcopenia early in the community, rather than in a hospital setting. This study developed a model to predict possible sarcopenia among older adults and to explore factors associated with possible sarcopenia. The study analyzed data from the Korean Frailty and Aging Cohort Study (KFACS) to create possible and non-possible sarcopenia classifications using the 2019 Asian Working Group for Sarcopenia criteria. Machine learning (ML) models, including Logistic Regression, Support Vector Machine, Decision Tree, and Random Forest, were developed to assess predictive performance, using the area under the curve (AUC) primarily. Analyses were conducted using Google Colab and JASP. A total of 1,761 participants (81.6±3.62 years, 46.7% male) were categorized into 500 possible sarcopenia (83.0±3.76 years, 34.4% men) and 1,261 non-possible sarcopenia (81.0±3.40 years, 51.6% men). The possible sarcopenia-prediction model built with a Random Forest algorithm achieved the highest AUC of 0.869. According to the feature-selection approach, walking speed, body mass index, age, instrumental activities of daily living, exhaustion, cognitive dysfunction, and quality of life were the most important predictors of possible sarcopenia in older adults across the four ML models. The Random Forest model was shown to be especially useful for community healthcare providers in detecting possible sarcopenia early in community-dwelling older adults. To reduce the impact of sarcopenia on the aging population, improved community screening that considers physical dysfunction as well as psychological aspects are needed.

